# Acceptance of assisted partner notification among HIV-positive adults with severe mental illness at a national referral hospital in Uganda: a cross-sectional study

**DOI:** 10.1186/s12913-024-10770-1

**Published:** 2024-03-08

**Authors:** Rachel Wangi Nante, Herbert Muyinda, John M. Kiweewa, Regina Ndagire, Emmanuel Ssendikwanawa, Kevin Ouma Ojiambo, Joanita Nangendo, Juliet Nakku, Fred C. Semitala

**Affiliations:** 1 Clinical Epidemiology Unit, Department of Medicine, College of Health Sciences, Makerere University, Kampala, Uganda; 2 Africa Centre for Systematic Reviews and Knowledge Translation, College of Health Sciences, Makerere University, Kampala, Uganda; 3 Child Health and Development Centre (CHDC), College of Health Sciences, Makerere University, Kampala, Uganda; 4 Education Department, Fairfield University, Fairfield, CT USA; 5 Butabika National Referral Mental Hospital, Kampala, Uganda; 6 Department of Medicine, School of Medicine, Makerere University College of Health Sciences, Kampala, Uganda; 7 Mulago Immune Suppression Syndrome Clinic, Mulago National Referral Hospital, Kampala, Uganda

**Keywords:** Assisted partner notification, HIV, Severe mental illness, Acceptance

## Abstract

**Background:**

HIV mostly affects people with severe mental illnesses (SMIs) than the general population. In 2015, the World Health Organization (WHO) introduced assisted partner notification (APN) as a strategy to increase HIV testing. Although research has demonstrated the effectiveness of APN in the general population, its use among people living with HIV (PLHIV) who have SMI is not well understood. This study sought to determine the acceptance of the APN strategy among PLHIV who had a diagnosis of SMI.

**Methods:**

This study used a cross-sectional study design that was retrospective to determine acceptance of APN among PLHIV with a documented diagnosis of SMI. We enrolled participants with a diagnosis of both HIV and SMI from August 2018 to January 2022, attending the HIV clinic at Butabika Hospital. We used pretested questionnaires to extract participants’ demographic and clinical data from their existing clinical charts, antiretroviral therapy (ART) registers and APN registers. We defined acceptance of APN as the number of PLHIV with SMI diagnoses who agreed to provide information about their sexual partners. We used modified Poisson regression analysis to assess the factors associated with the acceptance of APN.

**Results:**

A total of 125 participants were enrolled, of whom 83 (66.4%) were female. The median age was 30 (interquartile range (IQR) (25–34)), and 41 (33%) of them accepted APN (95% CI: 25.05–41.61). Receipt of at least three counselling sessions before enrollment in APN (aPR = 1.8, 95% CI: 1.72–1.98) was the most significant factor associated with increased acceptance of APN. Poor adherence to ART (aPR = 0.62, 95% CI: 0.54–0.80), being escorted to hospital by a distant relative (aPR = 0.55, 95% CI: 0.39–0.80), being married/cohabiting (aPR = 0.65, 95% CI: 0.60–0.81), and being a Seventh Day Adventist (SDA) (aPR = 0.53, 95% CI: 0.45–0.71) or Pentecostal (aPR = 0.44, 95% CI: 0.22–0.98) by faith were associated with reduced acceptance of APN.

**Conclusion and recommendation:**

The acceptance of APN is low among PLHIV with a diagnosis of SMI. More structured counselling would facilitate earlier identification of undiagnosed HIV-positive partners. We recommend a follow-up study to compare acceptance of APN among PLHIV with SMI and those without SMI.

## Background

By the end of 2022, an estimated 39 million people were living with HIV globally [1]. Two-thirds of these (25.6 million) are in Africa [2]. Research shows that HIV prevalence is higher among the population with severe mental illness (SMI) [3]. Within Africa, the prevalence of HIV among people with SMI is reported at 19.2%, which is more than triple what is reported in the general population at 6.2% and the greatest burden lies in sub-Saharan Africa [4]. Globally, mental health disorders are associated with a 4-10-fold increase in HIV acquisition and transmission within the general population [5]. In Uganda, survey reports showed an HIV prevalence of 11.3% among adult persons with SMI [6] compared to 5.8% in the adult general population [7]. In addition to increasing the burden of HIV, mental health conditions affect access to HIV prevention, testing, treatment and antiretroviral therapy (ART) adherence [8, 9].

In 2015, the World Health Organisation (WHO) implemented the assisted partner notification (APN) strategy to improve HIV testing [10]. APN is a process in which a trained service provider assists consenting PLHIV by disclosing their HIV status or discreetly notifying their sexual partners of their probable HIV infection and encouraging them to go for an HIV test [8, 11]. The strategy identifies people who were previously ignorant of their possible exposure to HIV. Guidelines on HIV testing propose APN as a critical technique for reaching and identifying at-risk groups to meet the UNAIDS 95-95-95 targets [8, 11]. Through APN, there has been reported progress in increasing HIV testing services in the general population providing a 30% yield [12]. However, people with SMI have not been given due attention considering the rise in their HIV burden [5]. SMI may prevent the anticipated benefit of APN, early HIV diagnosis and early initiation into care [8, 11]. Some of the behaviours that put individuals with severe mental illness at HIV risk include drinking alcohol before sex, having multiple sex partners, irregular condom use, and using “survival sex,” in which sex is exchanged for shelter, food, or money, as many persons with SMI are unable to work and are of a low socioeconomic status [13–17].

Regardless of the high risk of HIV in people with SMI, little is known about the acceptance of APN and the associated factors among this population. It is against this background that **t**his study sought to determine APN acceptance and associated factors among people living with HIV (PLHIV) who have a diagnosis of SMI at Butabika National Referral Mental Hospital in Uganda.

## Methods

### Study design and setting

We employed a retrospective cross-sectional study design.

The study was conducted at Butabika National Referral Mental Hospital, a public hospital in Uganda. It is a public psychiatric hospital, funded and administered by the Uganda Ministry of Health. The hospital is currently the only National Referral Mental Health Institute in the country. It is a teaching hospital that offers general and specialised mental health treatment. The hospital provides HIV testing services to all patients admitted with mental illnesses. It runs a regular outpatient HIV clinic that provides comprehensive HIV care to both patients with mental illness and PLHIV without mental illness who come from the neighboring community. HIV testing services are offered at three care points, including the outpatient department, inpatient department, and laboratory. Normally, individuals who are newly diagnosed with HIV and SMI are first admitted and managed on the ward until they are stable enough to participate in APN. Index HIV patients are enrolled in the APN program to enlist their sexual partners, who are subsequently approached for an HIV test. Results from the HIV test are recorded in the APN register. In addition, information about the number of sexual partners, the HIV status of those partners, if positive, and whether they are in care, is collected and recorded in the APN register.

### Study participants

We reviewed consecutive files of PLHIV with a diagnosis of SMI who were identified through any of the entry points and entered into the ART register. Patients were included in the study if they (1) had been diagnosed with HIV between 1st October 2018 and 31st January 2022 and (2) had a documented diagnosis of SMI. We defined SMI as a prolonged and recurrent mental disorder, that impairs activities of daily living and requires long-term treatment [18] and comprises bipolar disorder, major depressive disorder, psychosis, schizoaffective disorder, and schizophrenia [19]. We excluded participants who did not have outcomes recorded in the register.

### Sample size estimation and sampling technique

To determine the acceptance of APN, we estimated the sample size using the Leslie formula for a single proportion $\text{N}=\frac{{{\text{Z}}^{2}}_{\alpha /2}(1-\text{p})}{{\text{d}}^{2}}$, referring to a study conducted in Vietnam [20], where **Z-alpha** is the standard normal value corresponding to the set confidence level of 95% = **1.96, d** is the tolerable sampling error (precision) = **5%**, **p** is the estimated prevalence of APN acceptance in the study = **0.8** and **N** is the required sample size = 237. Since the number of individuals with HIV and SMI who were recorded in the APN register was small compared to the estimated sample size, we scaled it down using the formula $S=\frac{N}{1+N/Population\,size}$, where N is the estimated sample size = **237**, population size is the number of individuals with HIV and SMI recorded in the Butabika Hospital APN register by January 2022 = **150, and** S is the adjusted sample size = **92.** We considered a 10% increase in sample size to account for missing data. Therefore, the study needed a minimum of **102 participants** to answer the research question about the acceptance of APN.

To determine the factors associated with the acceptance of APN, we used the formula for 2 proportions referring to a study conducted in Kenya [21].

$$ N=\frac{{\left[{Z}_{\alpha /2}\sqrt{p\left(1-p\right)\left(\frac{1}{{q}_{1}}+\frac{1}{{q}_{2}}\right)+}{Z}_{\beta }\sqrt{{p}_{1}\left(1-{p}_{1}\right)\frac{1}{{q}_{1}}+{p}_{2}\left(1-{p}_{2}\right)\frac{1}{{q}_{2}}}\right]}^{2}}{{\left({p}_{1}-{p}_{2}\right)}^{2}},$$

where **q1** is the proportion of females who were eligible for APN = 0.642, **q2** is the proportion of males who were eligible for APN = 0.358, **Zα** is the standard normal value corresponding to the level of significance, for **α** = 0.05, **Zα** = 1.96, **Zβ** is the standard normal value corresponding to the power of the study, for power = 80%, Zβ = 0.84, **p1** is the proportion of females eligible for APN who accepted APN = 0.654, **p2 is the** proportion of males eligible for APN who accepted APN = 0.808, **p** = p1q1 + p2q2 and **N** is the required sample size. The minimum sample size required to determine acceptance and factors associated with acceptance of APN was 110. All files that did not have the outcome of interest were removed. All participants who met the study eligibility criteria were consecutively recruited into the study.

### Data collection

We developed a structured data abstraction tool and used it to abstract data from patient files. The tool was piloted for suitability on 10 randomly selected records of patients with severe mental illness recorded in the APN register and thereafter adjusted for errors accordingly to suit the study data. Data were obtained from patient’s medical records, which included the ART cards mental health files, and APN registers, using the data abstraction tool. Data were abstracted by the lead author and two enrolled nurses trained as research assistants. We collected data on age, sex, religion, marital status, relationship with next of kin (NOK), level of education, level of adherence to ART (good adherence to ART was defined as adherence ≥ 95%, fair as 80–94% and poor as < 80%), number of counselling sessions attended before a patient was offered APN services and documentation on drug abuse. In addition, we collected data on the acceptance of APN from the hospital APN register.

### Data analysis

Data were entered into Microsoft Excel, and analyses were performed using Stata, version 14 (Stata Corporation, College Station, Texas, USA). Categorical variables were summarised using frequencies and percentages and as medians with their interquartile ranges (IQR) for continuous variables. The primary outcome was the prevalence of acceptance of APN, which was defined as the proportion of individuals with HIV and documented diagnosis of SMI who provided information about their sexual partners. The numerator was HIV + individuals with a documented diagnosis of SMI who provided information about their sexual partner. The denominator was all participants with HIV and documented SMI who were eligible for APN between August 2018 and January 2022. A Poisson regression model with robust standard errors was used for bivariate analysis. Multivariate logistic regression was then performed to identify significant factors. A backward elimination method was used by removing the least likelihood variables until only significant factors emerged. Interaction was assessed using the chunk test, comparing likelihood ratios for full and reduced models. Similarly, confounding was assessed by comparing adjusted and unadjusted ORs, and a predictor variable with a difference of ≥ 10% was considered a confounder. Conclusions were drawn based on prevalence ratios (PRs) with their corresponding 95% confidence intervals and 0.05 level of significance.

## Results

### Characteristics of study participants

Between December 2021 and January 2022, we screened 139 patient files for eligibility. Of these, 125 (89.9%) were included in the study, and 14 files were excluded due to missing data on APN acceptance. Of the 125 participants, 66.4% were females with a median age of 30 (IQR 25–34). A third (34.2%) were single, 36.6% had attained primary education, 74.4% had a close relative as their next of kin and 31.3% had a documented history of drug abuse (Table 1).

### Prevalence of APN acceptance

Of the 125 study participants, 41 (32.8%) accepted APN. The majority (69%) were females, and the median (IQR) age was 29.5 (25–34) years. Only 48.6% were married, 78% had good adherence to ART, and 80% had received at least three counselling sessions before they were introduced to the APN.

### Factors associated with acceptance of APN

The factors significantly associated with the acceptance of APN included receiving at least 3 counselling sessions before they were offered APN, religion, marital status, level of adherence to ART and relationship with next of kin. Participants who had received counselling before they were offered APN services were more likely to accept APN than those who received less than three counselling sessions (aPR = 1.80, *P* = 0.018, 95% CI: 1.72–1.98). Participants who belonged to the Pentecostal or SDA religions were less likely to accept APN than those who belonged to the Catholic religion (Pentecostal, aPR = 0.44, *P* = 0.031, 95% CI; 0.22–0.98, SDA, aPR = 0.53, *P* = 0.02, 95% CI; 0.45–0.71). Similarly, married or cohabiting participants were less likely to accept APN than those who were unmarried (aPR = 0.65, *P* = 0.03, 95% CI; 0.60–0.81). In addition, participants who were escorted to the hospital by a distant relative were less likely to accept APN than those who were escorted by a close relative (aPR = 0.55, *P* < 0.001, 95% CI; 0.39–0.80). Finally, patients with poor adherence to ART were less likely to accept APN than those who had good adherence to ART (aPR = 0.62, *P* = 0.029, 95% CI: 0.54–0.80) (Table 2).

**Table 1:** Social demographic characteristics of people with HIV and severe mental illness at Butabika Hospital (*N* = 125)

Characteristic	Frequency, n (%)
Age (yrs)	
15–24	17(13.6)
25–34	53(42.4)
35–44	40(32.0)
> 44	15(12.0)
**Sex**	
Female	83(66.4)
Male	42(33.6)
**Religious affiliation#**	
Catholic	40(34.2)
Anglican	39(33.3)
Muslim	13(11.1)
Pentecostal	17(14.5)
SDA	8(6.8)
**Marital status#**	
Single/Never married	40(34.2)
Married/Cohabiting	39(33.3)
Divorced/Separated	20(17.1)
Widowed	5(4.3)
**Relationship with NOK**	
Close relative	93(74.4)
Distant relative	8(6.4)
Friend/Neighbor	24(19.2)
**Level of education#**	
No formal education	5(12.2)
Primary	15(36.6)
Secondary	13(31.7)
Post-secondary	8(19.5)
**Drug use#**	
Yes	36(31.3)
No	79(68.7)
**Level of adherence to ART#**	
Good (≥ 95%)	64(52.0)
Fair (80–94%)	23(18.7)
Poor (< 80%)	36(29.3)
**Received Counseling #**	
Yes	33(28.0)
No	85(72.0)

#Variables with missing data

**Table 2:** Factors associated with acceptance of APN among people with HIV and severe mental illness at Butabika Hospital (*N* = 125)

Variable	Category	n (%)	cPR(95% CI)	P value	aPR(95% CI)	P value
Age	25–34	16(30.2)	**1**			
	15–24	4(23.5)	0.95(0.79–1.15)	0.587		
	35–44	17(42.5)	1.10(0.95–1.26)	0.219		
	> 44	4(26.7)	0.97(0.80–1.19)	0.790		
Sex	Female	29(34.9)	1			
	Male	12(28.57)	0.95(0.84–1.09)	0.470		
Religion (*n* = 117)	Catholic	15(37.5)	1		1	
	Anglican	13(33.3)	0.97(0.83–1.13)	0.700	0.96(0.88–1.04)	0.297
	Pentecostal	5(29.4)	0.74(0.67–0.85)	0.056	0.44(0.22–0.98)	**0.031**
	SDA	3(37.5)	0.46(0.27–0.61)	**0.023**	0.53(0.45–0.71)	**0.022**
Marital status (*n* = 117)	Muslim	2(15.4)	0.84(0.69–1.03)	0.090	0.94(0.86–1.03)	0.164
	Married/Cohabiting	17(41.5)	1.16(1.01–1.34)	**0.036**	0.65(0.60–0.81)	**0.031**
	Divorced/Separated	4(20)	0.99(0.83–1.18)	0.884	0.97(0.91–1.03)	0.303
	Widowed	3(60)	1.32(0.99–1.75)	0.059	0.97(0.91–1.04)	0.071
Relationship with NOK	Close relative	34(36.6)	1		1	
	Friend/neighbour	6(25)	0.92(0.78–1.07)	0.268	0.32(0.20–0.53)	**< 0.001**
	Distant relative	1(12.5)	0.82(0.66–1.02)	0.080	0.55(0.39–0.80)	**< 0.001**
Level of education level (*n* = 41)	No formal education	2(40)	1.05(0.73–1.50)	0.790		
	Secondary	5(38.5)	1.04(0.80–1.35)	0.780		
	Post-secondary	3(37.5)	1.03(0.76–1.40)	0.844		
	No	54(68.3)	1			
History of drug abuse (*n* = 79)	Yes	25(31.7)	0.99(0.86–1.14)	0.907		
	Good (≥ 95%)	32(50)	1		1	
Level of Adherence to ART on the last visit (*n* = 123)	Fair (80–94%)	7(30.4)	0.87(0.74–1.03)	**0.100**	1.01(0.90–1.14)	0.87
	Poor (< 80%)	2(5.6)	0.70(0.63–0.78)	**< 0.001**	0.62(0.54–0.80)	**0.029**
	No	85	1		1	
Received Counseling (*n* = 118)	Yes	33(100)	1.87(1.78–1.97)	**< 0.001**	1.8(1.72–1.98)	**0.018**
	Yes	33(100)	1.87(1.78–1.97)	**< 0.001**	1.8(1.72–1.98)	**0.018**

## Discussion

This study assessed the acceptance of APN and associated factors among individuals with known diagnoses of HIV and SMI. We found very low evidence for APN acceptance, with only one-third of the participants utilizing this high-yield HIV testing strategy. This low acceptance could be because individuals who have an SMI are perceived not to be in a stable mental state to participate in APN, so health workers take some time to talk to them even when they are stable and fit to participate.

These findings of APN utilization are below the WHO target of 100% acceptance and the 80 and 89% acceptance reported in two previous retrospective studies conducted in Kenya among people with HIV without SMI [22, 23] respectively. We did not find any previous study on the acceptance of APN among PLHIV with SMI, hence, it is important to appreciate that given the two studies on APN acceptance did not include individuals with a diagnosis of SMI, this may account for such large differences in APN acceptance. However, some studies have noted that the acceptance of HIV services among people with SMI remains a major challenge [14, 24].

The study findings showed a significant positive association between routine counselling and APN acceptance. Individuals who received at least three counselling sessions before they were offered APN services were 80% more likely to accept APN compared to those who received less than 3 counselling sessions. This could be explained by the fact that during the counselling sessions, APN is one of the topics discussed, and counsellors ensure that by the time the sessions end, individuals willingly disclose their HIV status and have their sexual partners tested once approached. The findings concur with those of a qualitative study conducted in Barbados, which reported that engagement in counselling sessions before the introduction of the APN strategy improved the acceptance of APN [25].

Furthermore, the results revealed that poor adherence to ART (< 80%) was negatively associated with APN acceptance. Specifically, individuals who had poor adherence to ART were 38.2% less likely to accept APN. This low acceptance could be due to challenges attributable to mental illness including poor adherence to ART, poor retention among others, as reported in some studies. However, some studies indicate that the severity of a patient’s mental illness interferes with the ability to maintain scheduled ART treatment [27, 28]. The study also showed that affiliation with Pentecostal or SDA religions was associated with poor acceptance of APN. Over a quarter of Pentecostals and slightly more than half of SDAs accepted APN. The association between religious affiliation and APN acceptance is not clear, although there is research on the association between religion, faith, and spirituality in HIV prevention activities. It is, however, possible, as has been noted in other studies [29–31], that the beliefs and perceptions related to sexuality among these religious groups may negatively impact HIV prevention interventions. However, the general lack of literature on religion and the acceptance of APN calls for more studies. In addition, we found that APN acceptance was significantly lower among married individuals. Other studies have reported a low acceptance of HIV counselling and testing services among married couples [32, 33]. This finding was contrary to a cross-sectional study conducted in Tanzania by Lyamuya and colleagues which found that married participants were 5 times more likely to accept APN than those who were not [34]. Relatedly, widowhood had no significant association with the acceptance of APNs, which is similar to findings by Lyamulya and colleagues [34].

Being escorted by a distant relative or friend was less associated with accepting APN by 45% and 67.8%, respectively. This could be due to a lack of trust and fear of loss of confidentiality, which leads to fear of disclosing their private lives. It has been noted in some studies that stigma, discrimination and embarrassment associated with HIV infection are some of the reasons why PLHIV fear disclosing their HIV status [26, 35].

The study had some limitations; the study was based on secondary data, and we included some participants’ files with a few missing variables, although we ensured that all participants’ records contained data on the acceptance of APN. Furthermore, the total number of patients who were reported as declining APNs included those who were not told about APN, which could have overestimated the number that did not accept APN. Last, factors such as the type of partner (formal or informal), method of APN use (passive or active), and qualifications of health workers who offered APN were not included in the study; however, these factors have been considered important confounders in other studies [36]. The cross-sectional design of the study does not allow conclusions on a causal relationship. Regardless of these shortcomings, this is the first study to investigate APN acceptance among individuals with a dual diagnosis of HIV and SMI, despite research consistently showing a high prevalence of HIV among this population.

## Conclusion

There is a low acceptance of APN among PLHIV and a diagnosis of SMI in Uganda. Adequate counselling before APN service may improve its acceptance among patients with SMI; therefore, healthcare workers need to provide counselling to patients before they introduce them to APN. Additionally, factors such as marital status, religious affiliation, and nature of the relationship with those who accompany patients for treatment should be well addressed during the counselling sessions.

## Data Availability

The datasets used and/or analyzed during the current study are available from the corresponding author on request.

## Abbreviations

AIDS: Acquired Immune Deficiency Syndrome
APN: Assisted partner notification
ART: Antiretroviral therapy
FGD: Focus group discussion
HCW: Health Care Workers
HIV: Human Immune Virus
HIV+: HIV positive result
IDI: In-depth interview
IQR: interquartile ranges
KII: Key Informant Interview
NOK: Next of kin
PLHIV: Persons living with HIV
PR: prevalence ratio
RCT: Randomized controlled trial
SDA: Seventh-day Adventist
SMI: Severe mental illness
UNAIDS: Joint United Nations Programme on HIV/AIDS
WHO: World Health Organization
